# Thermochromic Polymer Film Sensors for Detection of Incipient Thermal Damage in Carbon Fiber–Epoxy Composites

**DOI:** 10.3390/s18051362

**Published:** 2018-04-27

**Authors:** Ryan Toivola, Sei-Hum Jang, Shawn Baker, Alex K. -Y. Jen, Brian D. Flinn

**Affiliations:** Department of Materials Science & Engineering, University of Washington, Seattle, WA 98195, USA; jangsh@uw.edu (S.-H.J.); shawnb1422@gmail.com (S.B.); ajen@uw.edu (A.K.-Y.J.); bflinn@uw.edu (B.D.F.)

**Keywords:** thermochromism, thermal damage detection, epoxy-matrix composites, fluorescence

## Abstract

Carbon fiber–epoxy composites have become prevalent in the aerospace industry where mechanical properties and light weight are at a premium. The significant non-destructive evaluation challenges of composites require new solutions, especially in detecting early-stage, or incipient, thermal damage. The initial stages of thermal damage are chemical rather than physical, and can cause significant reduction in mechanical properties well before physical damage becomes detectable in ultrasonic testing. Thermochromic fluorescent probe molecules have the potential to sense incipient thermal damage more accurately than traditional inspection methods. We have designed a molecule which transitions from a colorless, non-fluorescent state to a colorful, highly fluorescent state when exposed to temperature–time combinations that can cause damage in composites. Moreover, this molecule can be dispersed in a polymer film and attached to composite parts as a removable sensor. This work presents an evaluation of the sensor performance of this thermochromic film in comparison to ultrasonic C-scan as a method to detect incipient thermal damage in one of the most widely used carbon fiber–epoxy composite systems. Composite samples exposed to varying thermal exposures were used to evaluate the fluorescent thermal sensor films, and the results are compared to the results of ultrasonic imaging and short-beam shear tests for interlaminar shear strength.

## 1. Introduction

Composites present new and interesting challenges for non-destructive evaluation and inspection (NDE/NDI), given their inhomogeneity, anisotropy, opacity, complex chemical structure, and degradation behavior. With the increasing amount of composite structure in service, there is increased demand for development of NDE/NDI techniques and sensor technology to deal with the unique requirements these structures present [1,2,3,4]. 

Carbon fiber-reinforced epoxy composites (CFRE) have seen increasing use in aerospace and many other industries because of their highly desirable mechanical properties, light weight, and resistance to chemical/environmental degradation. One such composite is Toray’s T800 carbon fiber combined with 3900-2 toughened epoxy, which has a combination of high use temperature, mechanical strength, and toughness suitable for aerospace use [5,6,7,8]. T800/3900-2 is used to manufacture primary structure for Boeing’s 787 Dreamliner, and as such is one of the most important high-performance composite systems in the world [9].

An especially challenging problem in composite NDE/NDI is damage caused by thermal exposure. Moderate and severe thermal damage to CFRE can be detected visually or with standard in-service NDE/NDI techniques such as ultrasonic C-scan. However, the initial stages of thermal damage are often difficult to detect with these methods, and can cause reduction in the part’s mechanical properties, changes in glass transition temperature, matrix embrittlement, and matrix cracking [10]; these difficult-to-detect changes have been called Incipient Thermal Damage (ITD). Laboratory analysis techniques such as Raman spectroscopy, Fourier Transform Infrared Spectroscopy (FTIR) and Nuclear Magnetic Resonance (NMR) have had success at quantifying ITD by monitoring associated chemical changes in the epoxy matrix [11,12]; their applicability to in-service inspection is limited, however. Potential field-level inspection techniques based on diffuse reflectance FTIR and laser-induced fluorescence are in development, but have limitations in inspection area [10,13,14,15,16,17]. An adequate in-service NDE/NDI technique to detect ITD quickly and over a large area would be of great utility to the aerospace industry. 

The development of ITD in CFRE is chemical in nature and may involve hydrolysis, oxidation, and dehydration reactions of cured epoxy [11]. It is a kinetic process where both the temperature and duration of the exposure determine the extent of thermal damage—a long exposure at low temperature may cause similar damage to a short exposure at high temperature [18,19,20]. An ITD sensor for CFRE must behave not just as a ‘thermometer’ providing the current temperature, but as a ‘thermal history sensor’ accounting for the exposure time as well. Current sensor technology has few options that provide both of these features in a system suitable for rapid wide-area measurements. Thermocouples based on the Seebeck effect must be wired to a data recording device to provide the needed time–temperature data, which is not always possible in aerospace applications; thermocouples also only provide temperature data within a few microns of the bimetallic weld, and must be wired in an array to cover wide areas [21]. Temperature sensors based on fiber Bragg gratings show promise but must also be used as an array, with wiring requirements and wide-area application limitations similar to thermocouple arrays [3]. Commercially available thermal exposure labels, markers, and paints based on the melting point of a calibrated wax layer can only record the maximum temperature experienced by a surface without any time information [22]. 

Time–temperature indicator (TTI) sensors have had success in similar situations involving temperature-sensitive food products where spoilage extent depends on both exposure temperature and duration [23]. These indicator systems link the kinetics of food quality degradation with various time/temperature-sensitive property changes that cause quantifiable visible changes [24,25,26,27]. The TTI sensors are applied as decals and can be monitored with a single visual ‘read-out’. The most successful TTI systems are those which most closely match the quantifiable visible change kinetics with the food quality degradation kinetics. Our research goal is to design an ITD sensor system that can perform a similar service for composites. 

**Figure 1:**
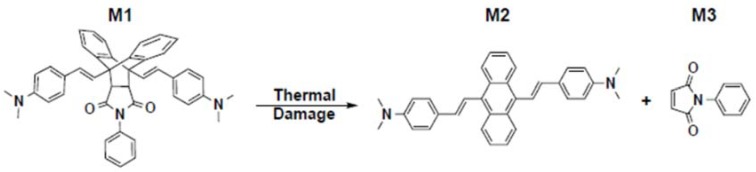
Molecule **M1** before and after damage-causing thermal exposures.

In previous work, we proposed using thermochromic fluorescent molecules dispersed into a host polymer as a new class of TTI sensor for ITD in CFRE [28]. Thermochromic fluorescence has been used as the basis for reversible “fluorescence thermometers” which cannot retain the necessary time information [21,29]. Irreversible thermal history sensors based on thermochromic fluorescence of lanthanide metals have inappropriate dynamic time and temperature ranges for aerospace composite applications [30,31]. With these challenges in mind, we developed a thermochromic fluorescent probe in which a highly fluorescent molecule, 9,10-Bis(p-dimethylaminostyryl)-anthracene (**M2**) is rendered non-fluorescent by the addition of N-phenyl maleimide (**M3**) to its anthracene core to form the initial molecule (**M1**) via the well-known [4+2] Diels–Alder reaction. Figure 1 shows the molecular structure and reaction in response to damage-causing thermal exposures. Exposing **M1** to sufficient heat causes a retro-Diels–Alder reaction which detaches **M3** and irreversibly returns the anthracene core to its fluorescent state in **M2** [32]. The activation kinetics of this **M1–M2** reaction match closely with reported kinetics of thermal damage formation in the T800/3900-2 CFRE system [28].

By incorporating this thermochromic molecule into a removable host polymer film, we have created a temperature film sensor suitable for wide-area application. The film can be applied and removed from parts after the time interval of interest using a suitable pressure-sensitive adhesive. The film can provide in situ wide-area visual ‘read-out’ based on the fluorescence changes due to the temperature driven **M1**–**M2** reaction. These changes can be observed by a user with a common UV lamp as optical excitation source; the film response can also be quantified using spectrometry, either in the field using portable equipment or in the lab after film removal using standard spectroscopic instrumentation. The host polymer we have chosen is poly (dimethyl siloxane) (PDMS), which has high optical transparency, thermal stability up to 350 °C, processing flexibility, and a low elastic modulus in its solid state allowing application of the film to curved structures. 

This study evaluates thermal sensor films of **M1**-PDMS as potential sensors for ITD in the important T800H/3900-2 CFRE system. A laboratory simulation of in-service thermal degradation of a CFRE panel using **M1**-PDMS films as monitoring ‘tabs’ attached to short-beam samples was performed. The fluorescent response of the **M1**-PDMS tabs, the interlaminar shear strength of the CFRE samples, and the ultrasonic scans of the samples before and after thermal exposure are presented and compared. 

## 2. Materials and Methods

### 2.1. Materials

The CFRE panels used in this study are formed from prepreg of Toray T800H fibers with 3900-2 resin in an 18 ply layup of a quasi-isotropic core with plain-weave 45° plies at the outside edges; the stacking sequence is [45w/[45/0/−45/90]_2s_/45w]. The panel was cured in an autoclave according to product specifications, at 177 °C for 2 h with 6.2 bar autoclave pressure. One side of the panel has a smooth tooled surface; the other has slight texture from the breather cloth/bagging film surface. 

The glass transition temperature (T_g_) of the panel was evaluated using differential scanning calorimetry (DSC) on a TA Instruments Q20 instrument, and also using dynamic mechanical analysis (DMA) on a Perkin Elmer DMA 7e instrument. The panel’s T_g_ was measured as 213 °C via DSC using the midpoint method of ASTM E1356 and 216 °C via DMA using the tan delta method of ASTM E1640 [33,34]. These results are presented in Appendix A. 

The host polymer for the sensor films is a PDMS, commercially available under the brand name Sylgard 184, acquired from Sigma Aldrich. It has two liquid components that are mixed and cured via a catalytic reaction into a transparent solid. 

The backing film used in this study is a transparent ethylene tetrafluoroethylene (ETFE) film, manufactured by Airtech International. 

The pressure-sensitive adhesive used to affix the sensor films to the backing film and to the carbon fiber–epoxy samples is a transparent silicone adhesive, brand named SilGrip PSA529. It is manufactured by Momentive Performance Chemicals. Toluene, used as a solvent, was obtained from Sigma Aldrich. 

### 2.2. Sample Preparation

Short-beam shear samples were cut from the CFRE panel using an Omax 2652 Precision Jetmachining Center and trimmed using a Pace PICO 150 low-speed saw with diamond blade to the dimensions dictated by ASTM D 2344 [35]. The resulting samples were 3.7 mm × 7.4 mm × ~22 mm for testing on a 14.8 mm span. 

Sensor films were prepared by dissolving powdered **M1** into toluene at a ratio of 1 mg **M1**:1 g toluene via ultrasonication for ~60 s. The **M1**-toluene solution was mixed into the liquid part A of PDMS at a ratio of 1:10 toluene:PDMS; the part B catalyst was subsequently added and mixed as instructed by the product data sheet. After mixing, samples were held under 28 mm Hg vacuum for ~10 min before pouring.

To construct sensor films, the solution was poured into a mold created by applying vacuum sealant tape to a rigid acrylic sheet that had been pre-treated with a silicone mold release. The solution was spread-cast over the surface and allowed to cure at room temperature for 48 h, after which the backing film was applied. 

The backing film for the sensors was prepared by applying a thin coat of PSA529 adhesive to the ETFE film using an Erichson Model 510 doctor blade coater. The adhesive was allowed to cure at room temperature for 24 h. The backing film was applied to the PDMS sensor film using a rolling pin and the combined film was refrigerated until use. The final film thickness was ~0.6 mm. 

### 2.3. Thermal Exposure

**Figure 2:**
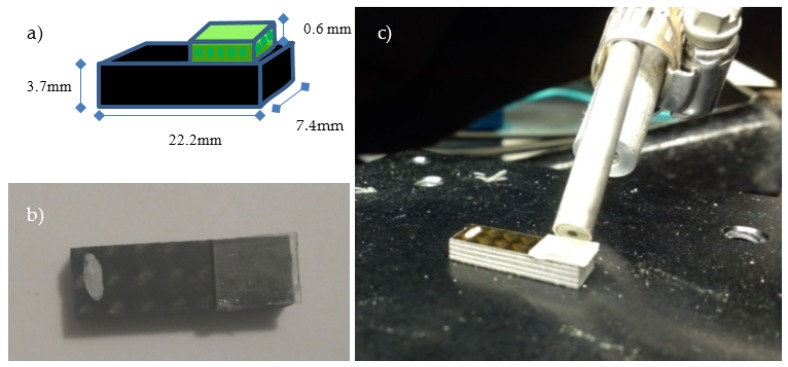
Carbon fiber reinforced epoxy short-beam samples with sensor ‘tabs’ attached. (**a**) Schematic of short-beam sample and tab; (**b**) Top-view image of short-beam sample and tab; (**c**) Side-view of short-beam sample and tab in fluorescent testing position.

A sensor film ‘tab’ was attached to each CFRE short-beam shear sample using a thin layer of PSA529 adhesive. Figure 2 has a diagram of the CFRE sample with ‘tab’ attached, along with a top-view image of the sample and a side view of the sample in position for fluorescent spectrum collection. Short-beam samples of CFRE were thermally exposed in sets of 5 samples for times of 10 min, 1 h and 6 h at each of several temperatures in a convection oven (Thermo Scientific HeraTherm OMH180). The samples were held with steel binder clips and suspended from a stainless steel rod to ensure even heat exposure on all surfaces. The rod and suspended samples were placed in the heated oven and allowed to reach the target temperature before starting the timed exposure, after which they were removed to room temperature and allowed to cool in air. One set of 5 samples was left at room temperature (labeled ‘no heat’) as a control. 

### 2.4. Non-Destructive Evaluation 

A standard NDE method for carbon fiber–epoxy composite parts in the industry is the ultrasonic scan [36,37]. A preferred method is water-coupled pulse-echo scanning, in which a sound wave from a probe passes through a sample and reflects back to the probe. Interfaces such as the front and back wall of a laminate, or any delamination or foreign bodies within the laminate, reflect strongly back to the probe; attenuation of the reflected signal can be an indication of porosity or microcracking. This method has been used to evaluate our short-beam samples before and after thermal exposures, and the ability of our thermal sensor films to detect ITD has been compared with the scans.

**Figure 3:**
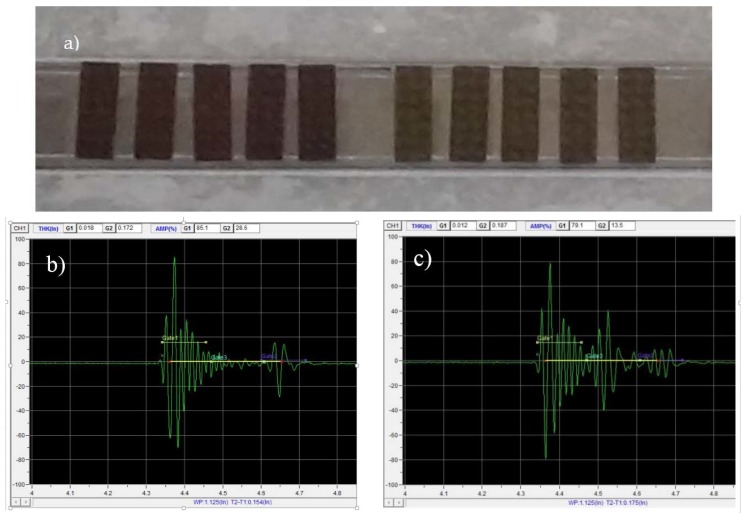
(**a**) Image of short-beam samples aligned in immersion tank for ultrasonic testing; (**b**) ‘A-scan’ data from undamaged area of a sample. (**c**) ‘A-scan’ data from damaged area of a sample.

Samples were evaluated using a Mistras water-immersion ultrasonic scanning instrument in pulse-echo mode with an Olympus V311 10 MHz ultrasonic transducer probe. Samples were aligned on an acrylic stair-step jig, and the probe was focused by bringing it into close contact with a sample then retracting it to the 25.4 mm focal distance. After focusing, the gain was increased until the front wall reflection signal reached 80% of the instrument maximum detection strength; digital amplitude correction was applied to enhance the back wall reflection signal to 80% of the maximum as well. The samples were scanned in unidirectional mode with speed setting 4 and resolution setting 0.01 in the Mistras UT NDIA software to collect the ‘A-scan’ data. To produce C-scans, the collected data was analyzed using ‘gates’ which cover the back wall reflection and the interior of the sample, in follow mode after the first echo from the sample front wall. The amplitude and time of flight of the peak in the signal that crossed the gate are plotted in C-scans. Figure 3 has a photo of the samples in the immersion tank on the jig, along with examples of the A-scans collected from an undamaged and a damaged sample.

### 2.5. Mechanical Testing

To test the performance of our sensor films as a detection method for ITD, it is necessary to choose a method of quantifying ITD for comparison. We have chosen the short-beam shear test as a measure of interlaminar shear strength (ILSS) for our method (ASTM D2344-16) [35]. This method has often been used in the literature as a way to quantify matrix properties in CFRE laminates under a variety of conditions including thermal, environmental and mechanical degradation [38,39,40]. Five samples from each thermal exposure were tested, and samples were visually checked after testing to ensure proper failure modes.

To determine the onset of ITD, we have chosen to use statistical methods. Student’s *t*-test is a procedure that compares two data sets to determine the likelihood that they have the same mean value; its output is a ‘*p*-value’ for the comparison that represents this probability [41]. We have chosen a *p*-value threshold for statistical significance of 0.01, corresponding to a 1% chance that the sample sets have the same mean ILSS value. The lowest temperature exposure that creates an ILSS change from control that crosses this threshold will be considered the onset exposure for ITD. 

To enable comparison to fluorescence measurements, the percent loss in ILSS was calculated using Equation (1):

(1) $$\%loss,\;ILSS=\;100*\frac{ILSS_{control}-ILSS_{sample}}{ILSS_{control}}$$

where *ILSS_control_* is the *ILSS* measured from ‘no heat’ samples.

### 2.6. Spectral Analysis and Imaging 

Fluorescent emission spectra for this work were collected with a Y-type 7-around-1 fiber optic fluorescence probe held at 45° to the sample surface and processed by a portable spectrometer (Stellarnet BLUE-Wave UVN 200). The excitation light was produced by a light-emitting diode (LED) source (Stellarnet SL1-LED) using a 390 nm LED bulb. Sensor films were cooled to room temperature after thermal exposure before spectra were collected. Fluorescent spectra have a small ‘divot’ at ~545 nm that is characteristic of the spectrometer during low-light emission measurements. 

Images of samples were collected using a Canon PowerShot ELPH 100 HS digital camera mounted on a small tripod. Natural light images were taken in ‘portrait’ mode with no flash using a white ceramic plate as background. Fluorescence images were collected in ‘long shutter’ mode using a 15 s exposure in a darkroom using a black felt cloth as background. The samples were illuminated using a software-controlled 3-LED bulb (MagicLight BLE), with color display set to blue only; the resulting ~465 nm illumination light was filtered by passing through a 436 nm ± 10 nm fluorescence filter (Edmund Optics) before reaching the sample. To isolate the fluorescent emission, the reflected illumination light was removed from the images using a 495 nm long pass filter (ThorLabs) attached to the camera lens housing. 

The sensor behavior of ITD films was quantified using the total emission intensity *I_total_*, as defined in Equation (2):

(2) $$I_{total}=\int_{450\;\mathrm{nm}}^{700\;\mathrm{nm}}I\left(\lambda \right)d\lambda$$

where *I(λ)* is the emission intensity at wavelength *λ*.

To enable a direct comparison between fluorescent spectra and short-beam strength test results, the percent gain in *I_total_* was determined using Equation (3):

(3) $$\%gain,\;I_{total}=\;100*\frac{\int_{450\;\mathrm{nm}}^{700\;\mathrm{nm}}I_{sample}\left(\lambda \right)d\lambda -\int_{450\;\mathrm{nm}}^{700\;\mathrm{nm}}I_{control}\left(\lambda \right)d\lambda }{\int_{450\;\mathrm{nm}}^{700\;\mathrm{nm}}I_{maximum}\left(\lambda \right)d\lambda -\int_{450\;\mathrm{nm}}^{700\;\mathrm{nm}}I_{control}\left(\lambda \right)d\lambda }.$$

where *I_maximum_* is determined from a spectrum from a film sample exposed to 260 °C for 1 h, which produced the maximum measured emission intensity, and *I_control_* is determined using a spectrum from a film sample with no thermal exposure.

The dynamic temperature range of the sensor can be determined by the intersection of tangent lines below, during and above the increase in *I_total_*. Details on this method can be found in Appendix B. 

## 3. Results

### 3.1. ITD of CFRE and Ultrasonic Testing Detection Threshold 

**Figure 4:**
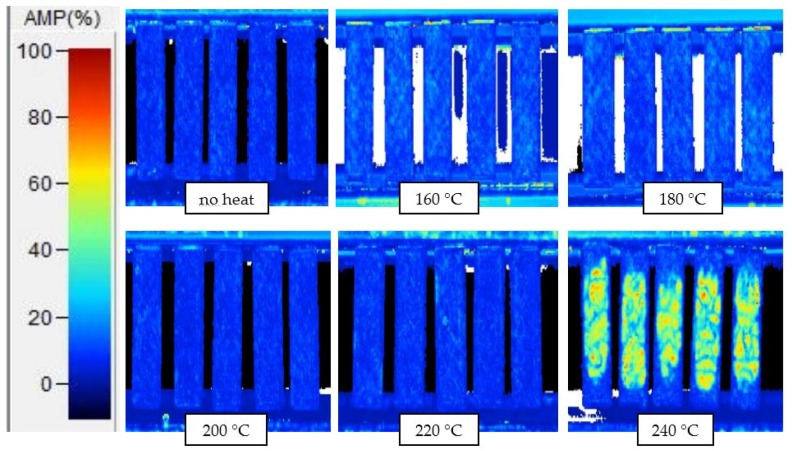
C-scans showing amplitude of interior reflections from short-beam samples exposed to various temperatures for 1 h.

Figure 4 shows C-scan images of samples after 1 h thermal exposures to various temperatures. In these C-scans, the amplitude of the peak signal crossing the interior gate is plotted; images of the amplitude of the ‘back-wall’ gate and time-of-flight of both gates are collected in Appendix C. Images of the C-scans created from the amplitude of the peak interior reflection of the 10 min and 6 h exposures are shown in Appendix D. 

Low amplitudes (blue) are indicative of coherent material with no reflective interfaces; high amplitudes (green/orange) indicate a reflective defect such as a delamination. Samples exposed to 240 °C for 1 h show significant delamination; samples exposed to temperatures of 220 °C and below do not show differences from non-exposed samples that can be detected in a simple analysis. Samples exposed for 6 h showed the same result—220 °C exposures produced no detectable damage but 240 °C exposures caused significant damage. Samples exposed for 10 min showed no detectable damage at exposures 240 °C and below, with significant damage observed at 260 °C exposures. These results establish the UT detection threshold for each exposure time. 

### 3.2. Mechanical Testing

**Figure 5:**
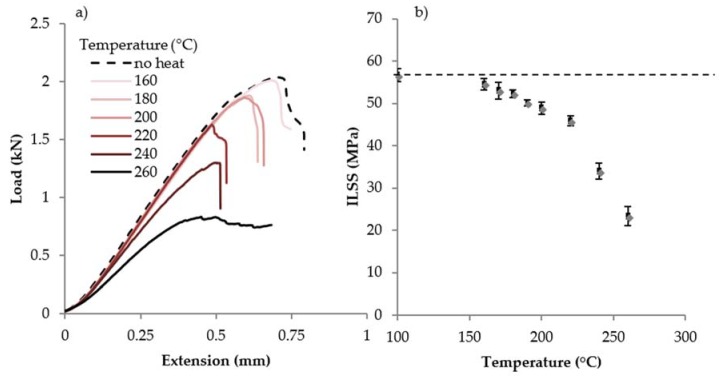
(**a**) Load vs. extension curves for short-beam samples of CFRE panel exposed to 1 h at various temperatures; (**b**) ILSS of samples exposed for 1 h. The dashed line in (**b**) represents the ILSS measured for samples with no thermal exposure.

**Figure 6:**
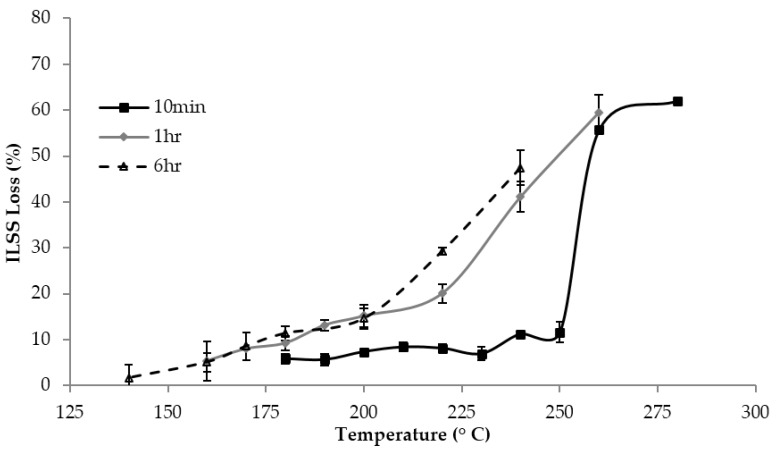
ILSS loss, in percent, vs. exposure temperature for samples due to thermal exposures of 10 min, 1 h and 6 h.

The results of mechanical testing for ILSS are shown in Figure 5 and Figure 6. Figure 5a shows load-displacement curves and Figure 5b shows the ILSS of samples tested after 1 h exposures at various temperatures. The decreasing load at failure is taken as an indication of the chemical damage to the matrix. There appears to be two distinct regions—at temperatures 160–220 °C, ILSS decreases slowly; above 220 °C, ILSS decreases more quickly. Figure 6 shows a plot of the ILSS loss as a percentage against temperature exposure from 10 min, 1 h and 6 h exposures, and for 10 min exposures, the temperature range where ILSS decreases slowly is extended to higher temperatures, while for 6 h exposures rapid ILSS loss begins earlier. A comparison with Figure 4 suggests the interpretation that the increase in ILSS loss rate corresponds to the onset of physical damage rather than chemical damage; this conclusion has been drawn by other researchers as well [10,14]. 

Appendix E has a table of the results of statistical significance testing using Student’s *t*-test. The loss in ILSS becomes statistically significant after 170 °C for 6 h exposures, and after 180 °C for 10 min exposures and 1 h exposures. These results in comparison to the ultrasonic C-scan results define the ITD region for each exposure time—180–260 °C for 10 min, 180–240 °C for 1 h, and 170–240 °C for 6 h.

The small but statistically significant reduction in ILSS at exposures as low as 170 °C is somewhat unexpected for this material, which has T_g_ above 210 °C as measured by DSC and DMA (see Appendix A). We attribute the decrease to stresses accumulated due to thermal gradients between the surface and interior of samples on heating and cooling, combined with mismatch in the coefficient of thermal expansion between fiber and matrix [42]. To produce nominally isothermal exposures, the heating and cooling rates involved in this study were in excess of 100 °C/min, likely high enough to prevent any dissipation of accumulated residual stresses on cooling to room temperature from the exposure temperature. Experiments using a much lower heating and cooling rate resulted in lower magnitudes of ILSS loss, and the loss due to 10 min of 180 °C exposure was statistically insignificant. These results are discussed in more detail in Appendix F of the Supporting Information. 

### 3.3. Fluorescent Activation of Sensors

**Figure 7:**
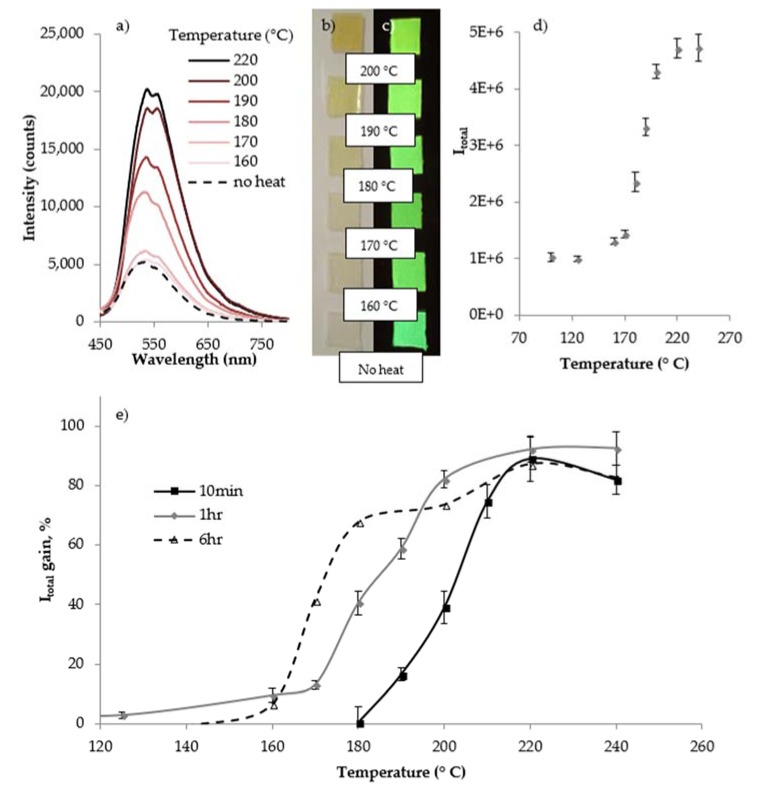
Fluorescent response of sensors to 1 h thermal exposures at various temperatures. (**a**) Emission spectra; (**b**) Ambient light images showing color change; (**c**) Fluorescent images showing intensity increase; (**d**) Integrated intensity values for 1 h exposure; (**e**) I_total_ gain % for 10 min, 1 h and 6 h exposures.

Figure 7 shows images and fluorescent spectra of the **M1**-PDMS tabs after 1 h thermal exposure. Figure 7a shows the emission spectra for different 1 h exposure temperatures. Figure 7b,c shows ambient and fluorescent images of samples after thermal exposure, labeled with the exposure temperature. The fluorescent intensity increase and color changes are clearly visible in the images. *I_total_* is plotted for exposure temperatures in Figure 7d. *I_total_* shows a sigmoidal increase as exposure temperature increases, approaching maximum activation near 220 °C. The dynamic range of the sensor (see Appendix B for details) is 169 to 204 °C for 1 h exposures. Figure 7e has a plot of the percent gain in I_total_ for the 10 min, 1 h and 6 h exposures. The visible and quantifiable dynamic range of the sensor begins at lower temperature for longer exposure. The next section compares the sensor’s dynamic range with the CFRE panel’s ILSS loss over these exposures.

### 3.4. Correlation between Sensor Activation and Mechanical Property Loss

The dynamic ranges of the sensor film for different times are collected in Table 1, along with the ITD region as determined from ILSS and ultrasonic C-scan data. The dynamic range of the **M1**-PDMS sensor film correlates well with the lower temperatures of the ITD region; in all cases the films’ intensity change has saturated before the highest exposures in the ITD region, and the film shows some activation before the onset of ITD at the 1 h and 6 h exposures. These may be desirable traits for a sensor as they would be a more conservative indicator for potential ITD-causing events. The sensor dynamic range for all exposure times begins at least 70 °C below the detection threshold of UT C-scan. 

**Figure 8:**
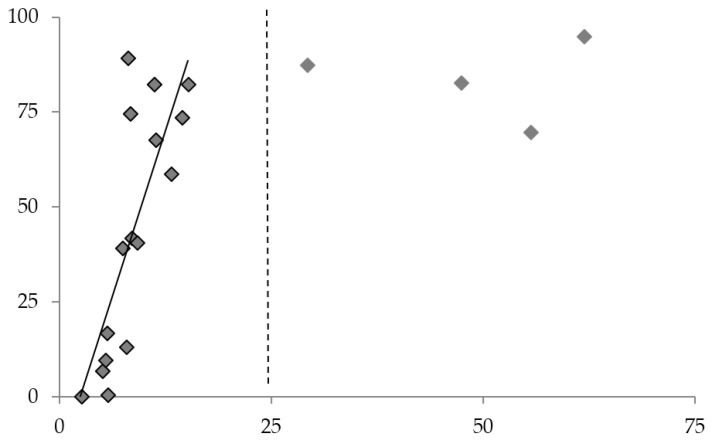
Percent I_total_ gain vs. percent ILSS loss for all exposures. Dashed line—exposures causing ILSS loss higher than this line were detectable via UT C-scan.

The comparison between ILSS loss and *I_total_* gain is another useful measure of **M1**-PDMS films as ITD sensors. The percent change of these two quantities is plotted against exposure temperature in Figure 8 for all three exposure temperatures. The sensor activation shows a good correlation for ILSS loss below ~25%, marked with a dashed line in Figure 8. Exposures that cause above 25% ILSS loss cause the sensor film intensity change to saturate, suggesting that the correlation is not as strong; however, all exposures that caused more than 25% ILSS loss in our study were detected with UT C-scan. These results strongly support the suitability of **M1**-PDMS films as sensors for ITD-causing thermal exposures in CFRE panels. 

As discussed in Section 3.2, these exposures and the fluorescent activation they cause are nominally isothermal, which required relatively high heating and cooling rates. Rapid heating and cooling likely contributed to the ILSS loss of the CFRE samples due to residual stresses or thermal shock from mismatch between the coefficients of thermal expansion of fibers and resin (see Appendix F). The effect of changes to heating and cooling rate on the extent of the **M1**-PDMS sensor film’s fluorescent activation and any resulting changes to its correlation with ILSS loss must be studied further. 

## 4. Conclusions

This work presents sensors made of a thermochromic fluorescent probe molecule **M1** dispersed in a PDMS host polymer to form a film suitable for wide-area thermal exposure measurements. The films are formed into removable “tabs” and used to detect incipient thermal damage-causing thermal exposures in a panel of the widely used T800/3900-2 carbon fiber–epoxy composite. Sensor activation was well correlated with the panel’s loss in interlaminar shear strength for the early stages of strength loss; this is an improvement over standard ultrasonic C-scan in detection capability for incipient thermal damage in this composite material. This research demonstrates the potential for sensor films based on thermochromic fluorescence which have advantages over existing sensor technologies such as thermocouple arrays and indicator paints in wide-area and temperature history data collection. Future studies will pursue the development of similar thermochromic sensor films with dynamic activation ranges calibrated for other temperature ranges of interest to aerospace and other industries including composite repair and structural health monitoring applications. 

## 5. Patents

US Patent number 9671386B2 “Detection of thermal damage of composites using molecular probes” resulted from the research in this project. 

## Figures and Tables

**Table 1:** Comparison of incipient thermal damage region of CFRE panel and dynamic range of M1-poly (dimethylsiloxane) sensor films.

Exposure Time	ITD Region (°C)	Sensor Dynamic Range (°C)
10 min	180–260	181–218
1 h	180–240	169–204
6 h	170–240	141–180

**Figure A1:**
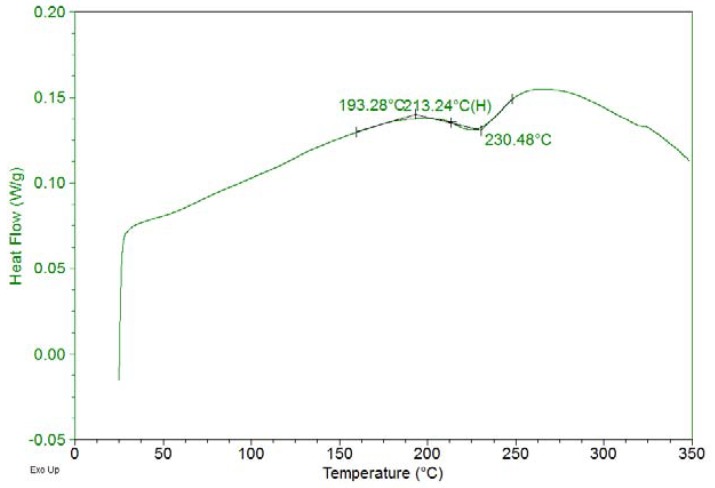
DSC trace of epoxy–matrix CFRE used in study.

**Figure A2:**
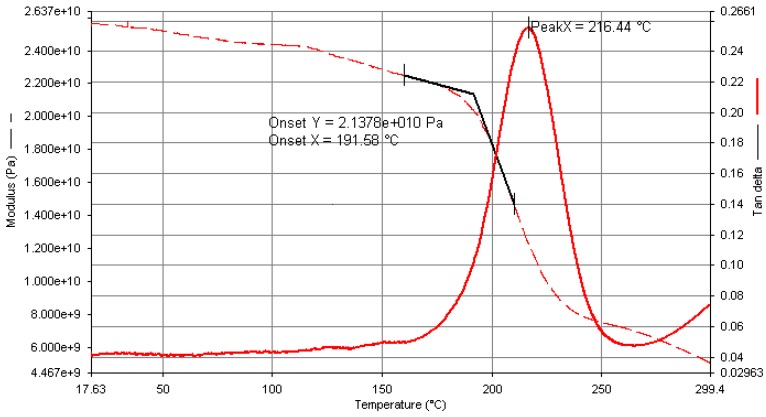
Storage modulus and tangent delta curves collected during DMA analysis of CFRE panel.

**Figure A3:**
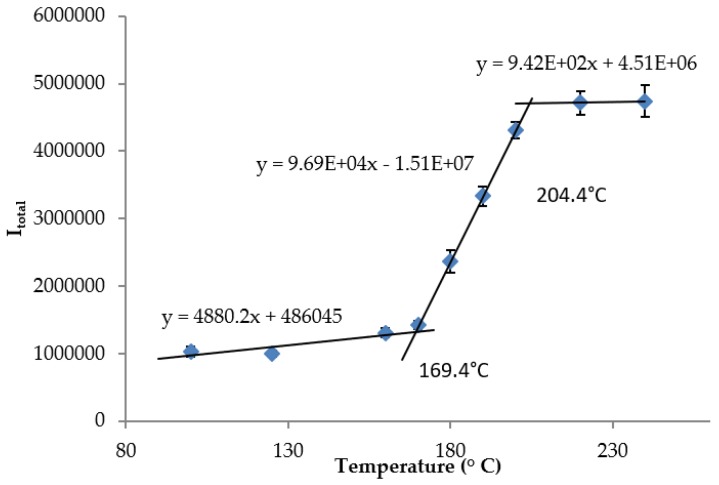
Sensor dynamic range calculations for 1 h thermal exposures.

**Figure A4:**
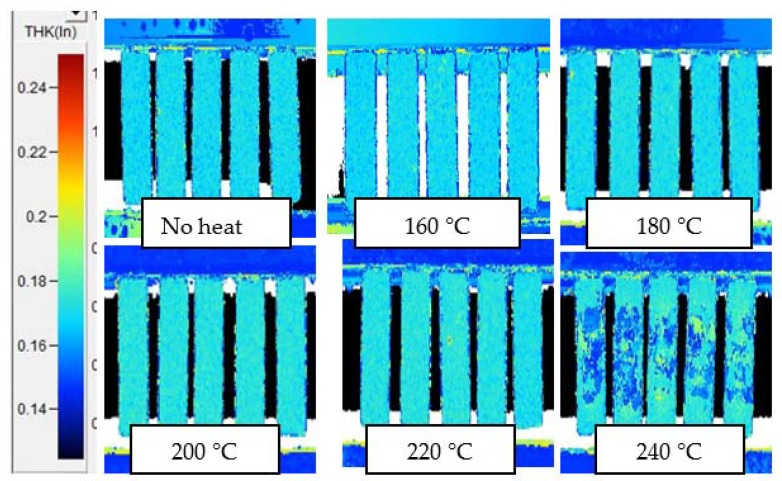
C-scan images of samples after 1 h exposure based on time-of-flight to maximum back wall echo.

**Figure A5:**
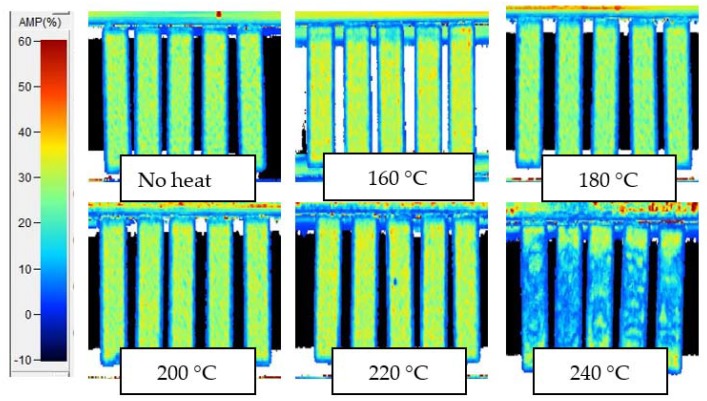
C-scan images of samples after 1 h exposure based on amplitude of back wall echo.

**Figure A6:**
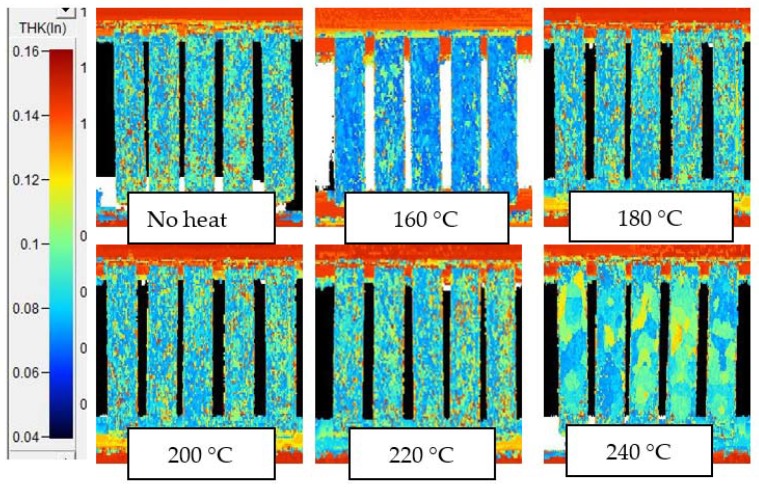
C-scan images of samples after 1 h exposure based on time-of-flight to maximum interior echo.

**Figure A7:**
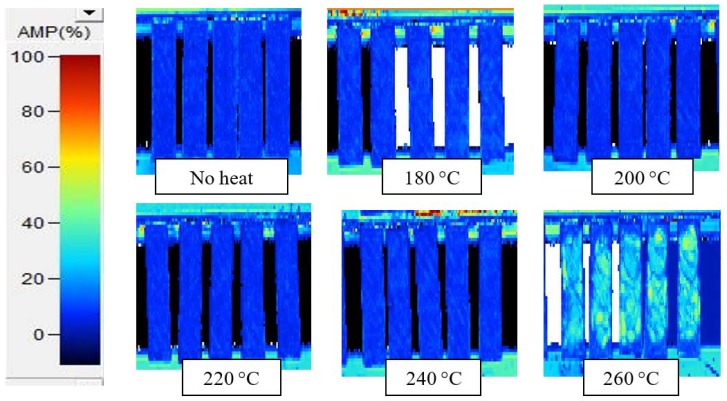
C-scans of samples after 10 min thermal exposures, using amplitude of maximum interior echo.

**Figure A8:**
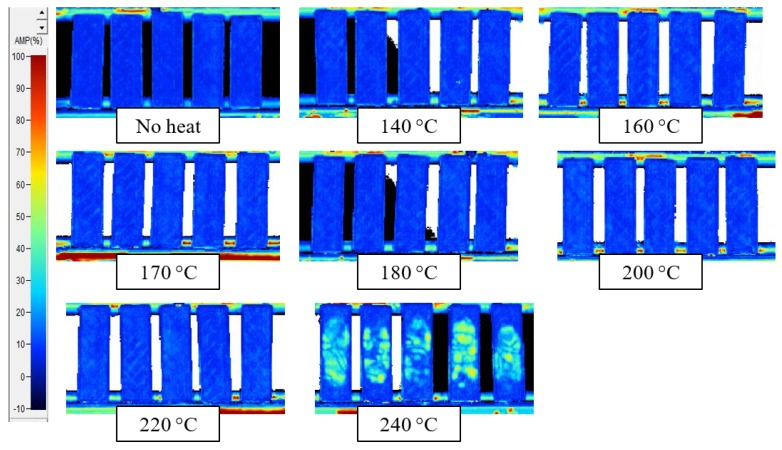
C-scans of samples after 6 h exposures, using amplitude of maximum interior echo.

**Figure A9:**
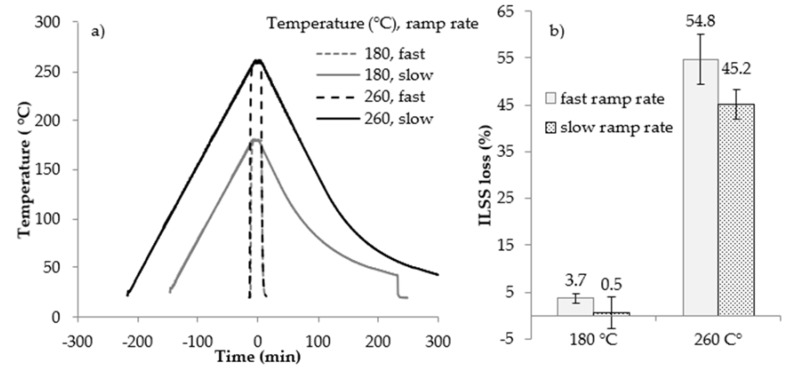
(**a**) Thermal exposure profile for exposures of 180 and 260 °C using fast and slow heating rates; (**b**) loss in ILSS, as percent of original, for 180 °C and 260 °C thermal exposures at fast and slow heating rates.

## Appendix A. Determination of Glass Transition Temperature for CFRE Panel

As described in the main text, the glass transition temperature (Tg) for the CFRE panel was determined using differential scanning calorimetry (DSC) and dynamic mechanical analysis (DMA). Figure A1 shows the DSC trace with analysis lines drawn by the ThermoFisher Universal Analysis software package. The T_g_ reported is the midpoint temperature, determined to be 213 °C by the software.

The panel’s T_g_ was also determined by dynamic mechanical analysis. Figure A2 has the storage modulus and tangent delta curves collected for a sample of the panel; in text the T_g_ is reported as the peak of the tangent delta curve, which occurs at 216 °C.

## Appendix B. Tangent Line Fitting for Dynamic Temperature Range for M1-PDMS Sensor

Fit lines were drawn using MS Excel through (Temperature, I_total_) data points above, below and during the sensor activation region. The temperature values of the interception points of these lines were taken as the beginning and end of the sensor dynamic range. Figure A3 shows the fit lines and interception points for 1 h exposures.

## Appendix E. *p*-Values Resulting from Comparison of ‘No Heat’ Samples’ ILSS with Thermally Exposed Samples’ ILSS Using Student’s *t*-Test.

**Temperature (°C)**	**10 min**	**1 h**	**6 h**
140			0.506
160		0.020498	0.05466
170		0.0277	0.0106
180	0.00579	9.01 × 10^−5^	1.865 × 10^−5^
190	0.00856		
200	0.00157	3.09 × 10^−5^	4.63 × 10^−5^
210	0.000342		
220	0.000475	8.94 × 10^−7^	1.26 × 10^−10^
230			0.00101
240	9.27 × 10^−5^	9.75 × 10^−7^	4.01 × 10^−6^
250			0.000247
260	3.92 × 10^−6^		
280	3.70 × 10^−10^		

## Appendix F. Comparison of High and Low Heating and Cooling Rates

In the main text, samples of a CFRE panel were exposed to elevated temperatures and subsequently tested for loss in interlaminar shear strength (ILSS). These exposures were designed to be isothermal. The samples were placed from RT into a hot oven, then exposed, then removed to cool in RT air. This exposure method resulted in heating and cooling rates of upwards of 100 °C/min. According to Hancox’s 1999 review article, (ref. [49] in main text) significant residual stresses can be accumulated in composite laminates due to thermal cycling at rapid heating and cooling rates. These residual stresses can be significant enough to cause delamination or microcracking, and industry practice during manufacture is to heat and cool at very slow rates to avoid these problems. As we observed ILSS loss in samples exposed to temperatures below their glass transition, which is not expected behavior, we briefly explored whether heating and cooling rates had any effect on ILSS loss for the same nominal thermal exposure.

We exposed samples to 180 °C and 260 °C for 10 min as in the main text, but placed samples into a cold oven and heated at an oven-controlled 2.5 °C/min instead of placing from RT into a hot oven. After the exposure, samples were cooled at an oven-controlled 2.5 °C/min to 40° C before removal to RT air, instead of direct removal to RT air. The resulting thermal exposure profile was monitored with surface mounted J-type thermocouples and recorded with LabVIEW as described in the main text. These thermal profiles are summarized and compared with those used in the main text in Figure A9a. The center of the isothermal exposure was set to t = 0 min to simplify comparison.

The samples were then tested for ILSS loss using the methods in the main text. Figure A9b plots percent ILSS loss for the two exposures and compares them with the results for rapid heating and cooling rates from the main text.

The slower heating and cooling rates produced significantly lower ILSS loss than the samples discussed in the main text. At 180 °C, the difference between original ILSS and post-exposure ILSS was not statistically significant for slow ramp rate exposure, but was significant for fast ramp rate exposure (see main text). At 260 °C, the ILSS loss was ~10% lower for slow ramp rate samples than for fast ramp rate samples. These results show that the observed losses in ILSS at unexpectedly low temperatures in the main text are likely the result of the high heating and cooling rates used in an attempt to create isothermal exposure conditions. Further study must be done to determine how thermal sensors will respond to non-isothermal heating and cooling scenarios.

## References

[1] Krishnamurthy S., Badcock R., Machavaram V., Fernando G. (2016). Monitoring Pre-Stressed Composites Using Optical Fibre Sensors. Sensors.

[2] Malik S.A., Wang L., Curtis P.T., Fernando G.F. (2016). Self-sensing composites: In-situ detection of fibre fracture. Sensors.

[3] Jenkins R.B., Joyce P., Mechtel D. (2017). Localized temperature variations in laser-irradiated composites with embedded fiber bragg grating sensors. Sensors.

[4] Zhang J., Li W., Cui H.-L., Shi C., Han X., Ma Y., Chen J., Chang T., Wei D., Zhang Y. (2016). Nondestructive Evaluation of Carbon Fiber Reinforced Polymer Composites Using Reflective Terahertz Imaging. Sensors.

[5] Hamerton I., Kratz J., Guo Q. (2018). The use of thermosets in modern aerospace applications. Thermosets: Structure, Properties, and Applications.

[6] Kageyama K., Kimpara I., Oshawa I., Hojo M., Kabashima S. (1995). Mode I amd Mode II Delamination Growth of Interlayer Toughened Carbon/Epoxy (T800/3900-2) Composite System. Compos. Mater. Fatigue Fract..

[7] Zhang J., Fox B.L. (2007). Manufacturing influence on the delamination fracture behavior of the T800H/3900-2 carbon fiber reinforced polymer composites. Mater. Manuf. Process..

[8] Swanson S.R., Qian Y. (1992). Muitiaxial characterization of T800/3900-2 carbon/epoxy composites. Compos. Sci. Technol..

[9] Griffiths B. (2005). Boeing sets pace for composites usage in large civil aircraft. High Perform. Compos..

[10] Dara I.H., Ankara A., Akovali G., Suzer S. (2005). Heat-damage assessment of carbon-fiber-reinforced polymer composites by diffuse reflectance infrared spectroscopy. J. Appl. Polym. Sci..

[11] Farquharson S., Bassilakis R., Ditaranto M.B., Haigis J.R., Smith W.W., Solomon P.R., Hartford E., Elleithy R., Ebeling T., Wallace J.F., Berthold J.W., Claus R.O., Marcus M.A., Rogowski R.S. (1994). Measurement of thermal degradation in epoxy composites by FT-Raman spectroscopy. SPIE Vol 2072: Fiber Optic Physical Sensors in Manufacturing and Transportation.

[12] Brady S.K., Conradi M.S., Vaccaro C.M. (2005). NMR detection of thermal damage in carbon fiber reinforced epoxy resins. J. Magn. Reson..

[13] Fisher W.G., Storey J.M.E., Sharp S.L., Janke C.J., Wachter E.A. (1995). Nondestructive Inspection of Graphite-Epoxy Composites for Heat Damage Using Laser-Induced Fluorescence. Appl. Spectrosc..

[14] Howie T., Tracey A., Flinn B. (2017). Composite Thermal Damage Measurement with Handheld Fourier Transform Infrared Spectroscopy.

[15] Rein A. (2014). Advanced Fourier Transform Infrared Spectroscopy for Analyzing Damage in Aircraft.

[16] Heckner S., Geistbeck M., Grosse C.U., Eibl S., Helwig A. (2015). FTIR Spectroscopy As a Nondestructive Testing Method for CFRP Surfaces in Aerospace. 7th International Symposium on NDT in Aerospace.

[17] Eibl S. (2017). Comparison of surface and bulk analytical techniques for the distinct quantification of a moderate thermal pre-load on a carbon fibre reinforced plastic material. Polym. Degrad. Stab..

[18] Musto P., Ragosta G., Russo P., Mascia L. (2001). Thermal-oxidative degradation of epoxy and epoxy-bismaleimide networks: Kinetics and mechanism. Macromol. Chem. Phys..

[19] Morgan R.J., Mones E.T. (1987). The cure reactions, network structure, and mechanical response of diaminodiphenylsulfone-cured tetraglycidyl 4,4′diaminodiphenyl methane epoxies. J. Appl. Polym. Sci..

[20] Bondzic S., Hodgkin J., Krstina J., Mardel J. (2006). Chemistry of thermal ageing in aerospace epoxy composites. J. Appl. Polym. Sci..

[21] Wang X., Wolfbeis O.S., Meier R.J. (2013). Luminescent probes and sensors for temperature. Chem. Soc. Rev..

[22] How It Works: Irreversible Temperature Indicator Labels. http://temperature-indicators.co.uk/articles/education/irreversible-temerature-indicators-educatio.htm.

[23] Taoukis P.S., Labuza T.P. (1989). Applicability of time temperature indicators as shelf life monitors of food products. J. Food Sci..

[24] Nuin M., Alfaro B., Cruz Z., Argarate N., George S., Le Marc Y., Olley J., Pin C. (2008). Modelling spoilage of fresh turbot and evaluation of a time-temperature integrator (TTI) label under fluctuating temperature. Int. J. Food Microbiol..

[25] Wanihsuksombat C., Hongtrakul V., Suppakul P. (2010). Development and characterization of a prototype of a lactic acid-based time-temperature indicator for monitoring food product quality. J. Food Eng..

[26] Fortin C., Goodwin H.L. Valuation of Temp-Time’s Fresh-Check^®^ Indicator on Perishable Food Products in Belgium. Proceedings of the Southern Agricultural Economics Association Annual Meeting.

[27] Wu D., Wang Y., Chen J., Ye X., Wu Q., Liu D., Ding T. (2013). Preliminary study on time-temperature indicator (TTI) system based on urease. Food Control.

[28] Toivola R., Howie T., Yang J., Lai P., Shi Z., Jang S.-H., Jen A.K.-Y., Flinn B.D. (2017). Highly Sensitive Thermal Damage Sensors for Polymer Composites: Time Temperature Indicator Based on Thermochromic Fluorescence Turn-On Response. Smart Mater. Struct..

[29] Khan M.R.R., Kang S.W. (2016). Highly sensitive temperature sensors based on fiber-optic PWM and capacitance variation using thermochromic sensing membrane. Sensors.

[30] Rabhiou A., Feist J., Kempf A., Skinner S., Heyes A. (2011). Phosphorescent thermal history sensors. Sens. Actuators A Phys..

[31] Talghader J.J., Mah M.L., Yukihara E.G., Coleman A.C. (2016). Thermoluminescent microparticle thermal history sensors. Microsyst. Nanoeng..

[32] Shi Z., Liang W., Luo J., Huang S., Polishak B.M., Li X., Younkin T.R., Block B.A., Jen A.K.Y. (2010). Tuning the kinetics and energetics of Diels-Alder cycloaddition reactions to improve poling efficiency and thermal stability of high-temperature cross-linked electro-optic polymers. Chem. Mater..

[33] ASTM International (2008). ASTM E1356 Standard Test Method for Assignment of the Glass Transition Temperatures by Differential Scanning Calorimetry.

[34] ASTM International (2013). ASTM E1640 Standard Test Method for Assignment of the Glass Transition Temperature by Dynamic Mechanical Analysis.

[35] ASTM International (2016). ASTM D2344-16: Standard Test Method for Short-Beam Strength of Polymer Matrix Composite Materials.

[36] Agarwal B.D., Broutman L.J. (1990). Analysis and Performance of Fiber Composites.

[37] Henneke E.G., Summerscales J. (1990). Ultrasonic Nondestructive Evaluation of Advanced Composites. Non-Destructive Testing of Fibre-Reinforced Plastics Composites.

[38] Mouritz A.P., Gallagher J., Goodwin A.A. (1997). Flexural strength and interlaminar shear strength of stitched GRP laminates following repeated impacts. Compos. Sci. Technol..

[39] Tsenoglou C.J., Pavlidou S., Papaspyrides C.D. (2006). Evaluation of interfacial relaxation due to water absorption in fiber-polymer composites. Compos. Sci. Technol..

[40] Shin K.B., Kim C.G., Hong C.S., Lee H.H. (2000). Prediction of failure thermal cycles in graphite/epoxy composite materials under simulated low earth orbit environments. Compos. Part B Eng..

[41] Raju T.N. (2005). William Sealy Gosset and William A. Silverman: Two “students” of science. Pediatrics.

[42] Hancox N.L. (1998). Thermal effects on polymer matrix composites: Part 1. Thermal cycling. Mater. Des..

